# Intact Cohesion, Anaphase, and Chromosome Segregation in Human Cells Harboring Tumor-Derived Mutations in *STAG2*

**DOI:** 10.1371/journal.pgen.1005865

**Published:** 2016-02-12

**Authors:** Jung-Sik Kim, Xiaoyuan He, Bernardo Orr, Gordana Wutz, Victoria Hill, Jan-Michael Peters, Duane A. Compton, Todd Waldman

**Affiliations:** 1 Lombardi Comprehensive Cancer Center, Georgetown University School of Medicine, Washington, D.C, United States of America; 2 Department of Biochemistry, Geisel School of Medicine at Dartmouth, Hanover, New Hampshire, United States of America; 3 Research Institute of Molecular Pathology (IMP), Vienna, Austria; Duke University, UNITED STATES

## Abstract

Somatic mutations of the cohesin complex subunit *STAG2* are present in diverse tumor types. We and others have shown that *STAG2* inactivation can lead to loss of sister chromatid cohesion and alterations in chromosome copy number in experimental systems. However, studies of naturally occurring human tumors have demonstrated little, if any, correlation between *STAG2* mutational status and aneuploidy, and have further shown that *STAG2*-deficient tumors are often euploid. In an effort to provide insight into these discrepancies, here we analyze the effect of tumor-derived *STAG2* mutations on the protein composition of cohesin and the expected mitotic phenotypes of *STAG2* mutation. We find that many mutant STAG2 proteins retain their ability to interact with cohesin; however, the presence of mutant *STAG2* resulted in a reduction in the ability of regulatory subunits WAPL, PDS5A, and PDS5B to interact with the core cohesin ring. Using AAV-mediated gene targeting, we then introduced nine tumor-derived mutations into the endogenous allele of *STAG2* in cultured human cells. While all nonsense mutations led to defects in sister chromatid cohesion and a subset induced anaphase defects, missense mutations behaved like wild-type in these assays. Furthermore, only one of nine tumor-derived mutations tested induced overt alterations in chromosome counts. These data indicate that not all tumor-derived *STAG2* mutations confer defects in cohesion, chromosome segregation, and ploidy, suggesting that there are likely to be other functional effects of *STAG2* inactivation in human cancer cells that are relevant to cancer pathogenesis.

## Author Summary

Mutations of the *STAG2* gene are common in several types of adult and pediatric cancers. In fact, *STAG2* is one of only 12 genes known to be significantly mutated in four of more types of cancer. The *STAG2* gene encodes a protein component of the “cohesin complex,” a ring-like structure that binds chromosomes together (e.g., “coheres” them) until the cohesin complex is degraded during cell division, allowing replicated chromosomes to separate normally to the two new cells. The cohesin complex also plays important roles in other cellular processes including turning genes on and off, and in repairing damaged genes. Here we analyze the effect of cancer-causing mutations in *STAG2* on its ability regulate the separation of chromosomes during cell division.

## Introduction

Cohesin is a multiprotein complex comprised of four primary subunits (SMC1A, SMC3, RAD21, and either STAG1 or STAG2) and four regulatory subunits (WAPL, CDCA5, and PDS5A or PDS5B) that is responsible for sister chromatid cohesion, regulation of gene expression, DNA repair, and other phenotypes [1,2]. Somatic mutations of cohesin subunits are common in a wide range of pediatric and adult cancers [3,4]. STAG2 (also known as SA2) is the most commonly mutated subunit, presumably in part because the *STAG2* gene is located on the X chromosome and therefore requires only a single mutational event to be inactivated [5]. Approximately 85% of tumor-derived *STAG2* mutations lead to premature truncation of the encoded protein, whereas approximately ~15% are missense mutations.

*STAG2* mutations are particularly common in bladder cancer (present in 30–40% of the most common non-muscle invasive tumors), Ewing sarcoma (present in ~25% of tumors), and myeloid leukemia (present in ~8% of tumors), and are also present in glioblastoma multiforme (GBM), melanoma, and other tumor types [6,7,8,9,10,11,12,13,14,15]. Highlighting the importance of *STAG2* as a cancer gene, in 2014 The Cancer Genome Atlas identified *STAG2* as one of only 12 genes that are significantly mutated in four or more human cancer types (the others were *TP53*, *PIK3CA*, *PTEN*, *RB1*, *KRAS*, *NRAS*, *BRAF*, *CDKN2A*, *FBXW7*, *ARID1A* and *KMT2D*; [16]). Among the other components of cohesin, *RAD21* is the most commonly mutated subunit, with mutations of *SMC1A* and *SMC3* also present in a subset of tumors. In addition to the frequent mutations in human tumors, the role of *STAG2* inactivation in cancer pathogenesis is also highlighted by the fact that it is commonly altered in transposon-mediated tumorigenesis in mouse model systems [17,18].

The mechanism(s) through which cohesin gene mutations confer a selective advantage to cancer cells is controversial. In our initial studies identifying *STAG2* mutations in cancer, we demonstrated using isogenic human cultured cell systems that *STAG2* mutations can lead to alterations of chromosome counts and aneuploidy [5,6]. These findings were consistent with other observations in yeast, mice, and other model systems indicating that mutations in cohesin subunits lead to chromosomal non-disjunction and aneuploidy [19,20,21,22]. However, more recent studies of naturally occurring human tumors have demonstrated either weak or no correlations between the presence of cohesin gene mutations and aneuploidy [8]. Furthermore, a subset of naturally occurring human tumors harboring cohesin gene mutations are euploid.

These conflicting data are likely in part attributable to the paucity of currently available functional data on tumor-derived *STAG2* mutations. For example, our reported functional studies of tumor-derived *STAG2* mutations have been primarily limited to two truncating mutations present in the human H4 and 42MGBA GBM cell lines (which were corrected by human somatic cell gene targeting). Others have demonstrated that myeloid leukemia cell lines harboring specific cohesin gene mutations have reduced levels of chromatin-bound cohesin compared to non-isogenic myeloid leukemia cells harboring wild type cohesin genes [10]. These authors further showed that ectopic expression of wild-type *STAG2* can lead to growth suppression of myeloid leukemia cells harboring specific endogenous mutations of *STAG2*.

Other work has focused on the functional consequences of *STAG2* depletion in mammalian cells, without testing individual mutations. For example, it has recently been shown that transient depletion of *STAG2* in human cells leads to a significant increase in errors in chromosome segregation due to aberrant kinetochore-microtubule attachments [23]. Furthermore, we and others have shown that stable depletion of *STAG2* in bladder cancer cells leads to alterations in chromosome counts [6,24].

Here we report a comprehensive analysis of the effects of tumor-derived *STAG2* mutations on the composition of cohesin and on mitotic phenotypes generally attributed to cohesin inactivation. We find that many mutant STAG2 proteins retain the ability to interact with cohesin, but result in a generalized reduction in levels of core cohesin subunits and in the ability of PDS5A, PDS5B, and WAPL to interact with the core cohesin ring. We then created and studied isogenic sets of human cells in which the endogenous allele of *STAG2* was modified via the addition of nine different tumor-derived mutations. While all nonsense mutations led to abrogation of sister chromatid cohesion and some nonsense mutations led to anaphase defects, tumor-derived missense mutations behaved like wild-type in these assays. Furthermore, few of the cell lines demonstrated the expected alterations in chromosome counts.

## Results

### Ectopic expression of tumor-derived *STAG2* mutants in human cells

**Fig 1:**
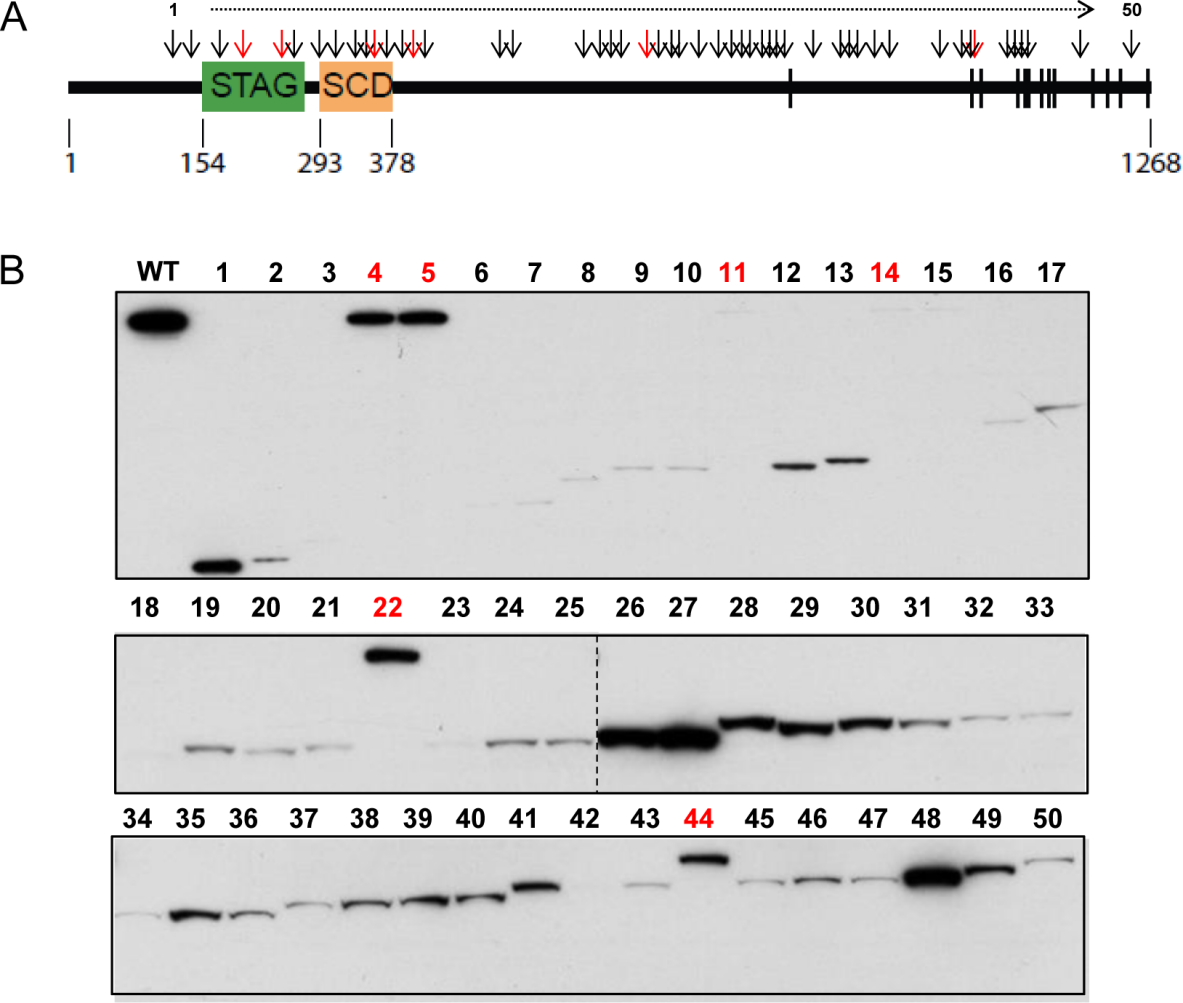
Expression of tumor-derived mutations of *STAG2* in human cells. (A) Mutations tested are depicted by arrows and described in detail in S1 Table. Truncating mutations are in black, and missense mutations are in red. STAG, stromal antigen domain; SCD, stromal in conserved domain. Phosphorylation sites are denoted by vertical bars. (B) 293T cells were transfected with wild-type and mutant epitope-tagged *STAG2* expression vectors, harvested in 1% NP40 lysis buffer, and their expression measured via immunoblotting with FLAG antibodies.

To express tumor-derived *STAG2* mutations in human cells, we created a full length human *STAG2* expression vector with a 1x FLAG/Streptavidin Binding Peptide (SBP) dual epitope tag at the amino terminus. This human *STAG2* expression vector corresponds to transcript CCDS43990. This new cDNA expression vector differs from the *STAG2* expression vector used in previous studies (transcript CCDS14607), which was a naturally-occurring splice variant missing the 37 amino acids encoded by exon 30. Transfection of the new, dual epitope-tagged expression vector into 293T cells led to expression of the tagged *STAG2* cDNA (Fig 1).

In naturally occurring human tumors, ~85% of *STAG2* mutations are truncating and ~15% are missense, with mutations spread roughly evenly throughout the gene [3]. There is also a minor mutational hotspot in Ewing sarcoma (R216X; ref. 13), found in ~20% of Ewing sarcoma tumors harboring mutations of *STAG2*. To model these mutations, we used site-directed mutagenesis to introduce 50 tumor-derived mutations into the cloned cDNA. The mutations were derived from numerous tumor types and were spread evenly throughout the gene (Fig 1A and S1 Table). These constructs were transfected into 293T cells, and their expression measured by Western blotting with FLAG antibodies. As depicted in Fig 1B, most of the truncating mutations were expressed very poorly, possibly due to nonsense mediated decay of the encoded transcript. Four missense mutations (#4,5,22,44) were expressed at levels equivalent to wild-type protein.

### Many tumor-derived mutant STAG2 proteins retain the ability to interact with the cohesin complex

**Fig 2:**
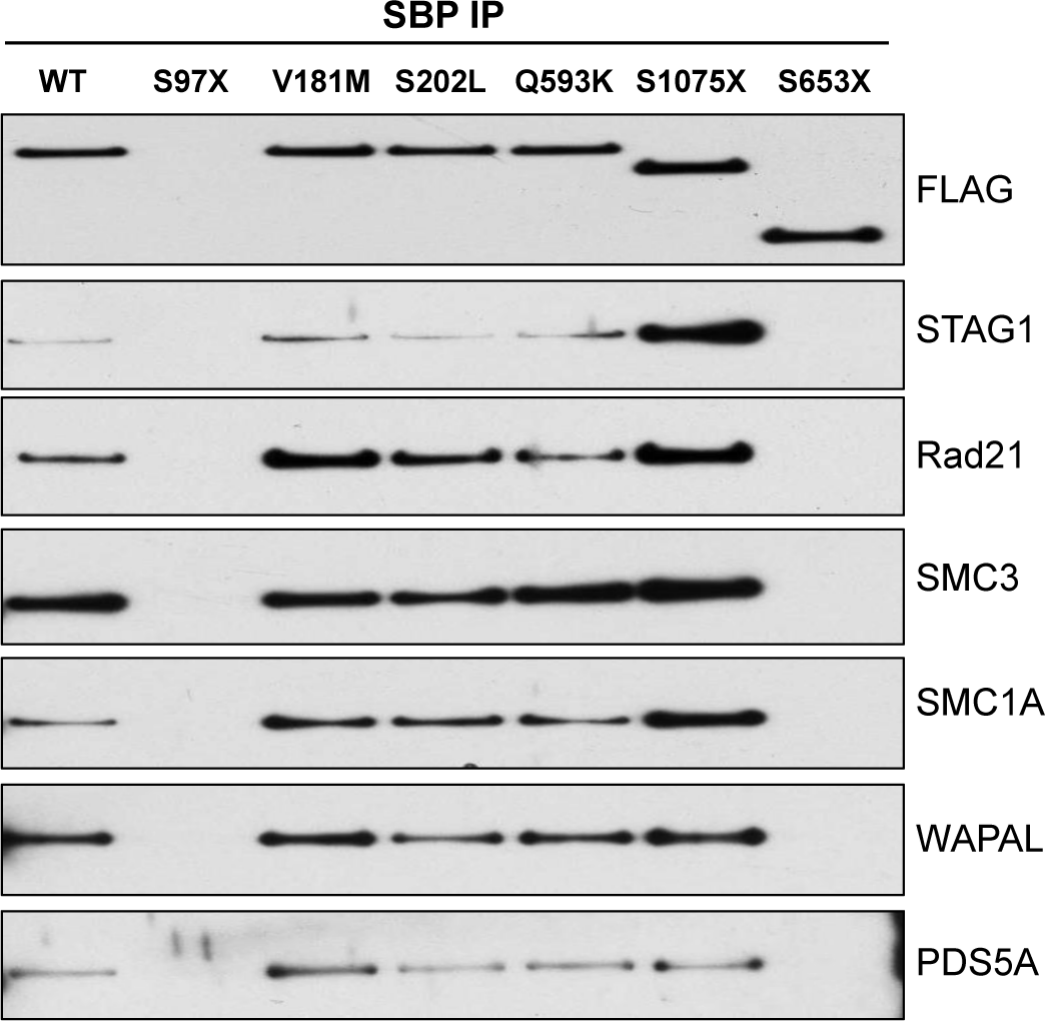
Interaction of mutant STAG2 proteins with cohesin. 293T cells were transfected with wild-type and mutant *STAG2* epitope tagged expression vectors and harvested in 1% NP40 lysis buffer. Immunoprecipitation was performed with streptavidin beads, and immunoblotting was performed with the antibodies indicated. Mutations tested include S97X, bladder cancer; V181M, myeloid leukemia; S202L, bladder cancer; Q593K, bladder cancer; S653X, GBM; S1075X, bladder cancer.

Arguably the most straightforward hypothesis regarding the loss of activity caused by tumor-derived *STAG2* mutations is that the mutations abolish the ability of the encoded STAG2 protein to interact with the rest of the cohesin complex. To test this, we measured the interaction of three missense mutations and two nonsense mutations with cohesin subunits SMC1A, SMC3, RAD21, STAG2, STAG1, WAPL, and PDS5A by immunoprecipitation (IP)—Western blotting. As depicted in Fig 2, each of the three proteins encoded by *STAG2* genes harboring tumor-derived missense mutations retained its ability to interact with cohesin. Similarly, a truncated protein encoded by a *STAG2* gene harboring a tumor-derived late truncating mutation (S1075X) also retained its ability to interact with cohesin. In contrast, the STAG2 protein encoded by a gene with an earlier truncating mutation (S653X) lost the ability to interact with the rest of the cohesin complex.

**Fig 3:**
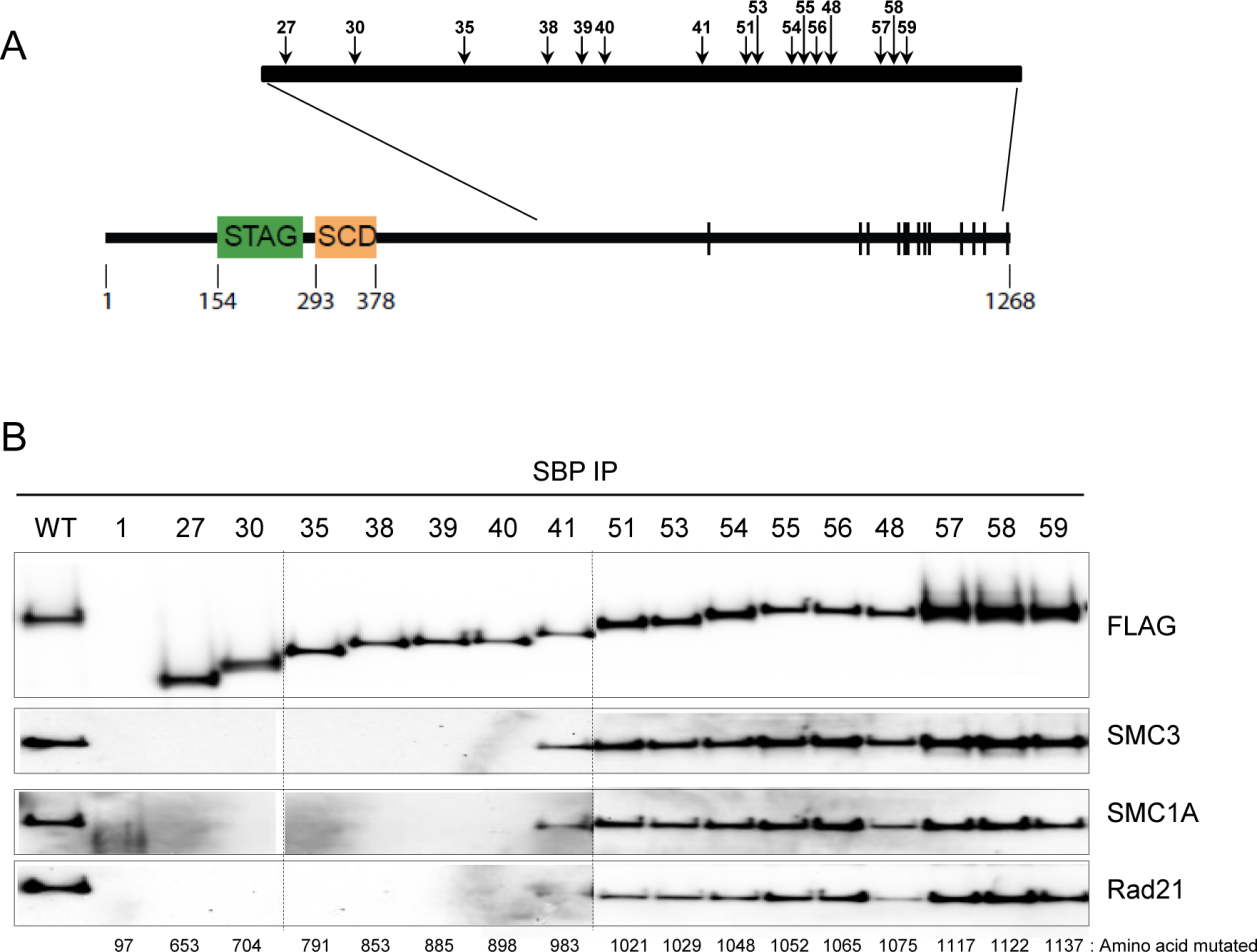
Identification of the boundary for interaction of STAG2 with cohesin. The mutations tested are depicted in (A) and described in detail in S1 Table. STAG, stromal antigen domain; SCD, stromal in conserved domain. Phosphorylation sites are denoted by vertical bars. (B) 293T cells were transfected with wild-type and mutant epitope tagged expression vectors and harvested in 1% NP40 lysis buffer. Immunoprecipitation was performed with streptavidin beads, and immunoblotting was performed with the antibodies indicated.

We next determined the boundary in STAG2 protein at which tumor-derived truncating mutations retained their ability to interact with cohesin. To do this, we created nine additional tumor-derived truncating mutations in *STAG2* from amino acid 1021 to amino acid 1137 in the cloned *STAG2* expression vector (#51–59; Fig 3A, S1 Table), transfected 293T cells, and performed IP-Western blotting. As depicted in Fig 3B, amino acids 983 to 1268 are dispensable for the interaction with cohesin, corresponding roughly to the beginning of the regulatory phosphorylation sites in the STAG2 protein [25]. These results are concordant with data from the recently published crystal structure of STAG2, which shows that STAG2 interacts with cohesin via an extensive interface with RAD21 that spans nearly the entire length of STAG2 and that is not disrupted by missense mutations targeting the interface [26]. Taken together, these data demonstrate that tumor-derived mutations of *STAG2* do not uniformly lose the ability to interact with cohesin, indicating that at least some tumor-derived mutations of *STAG2* must affect a key function of STAG2 other than its ability to interact with cohesin.

### Effects of *STAG2* mutations on the composition of cohesin

Having shown that many *STAG2* mutations do not abrogate the ability of STAG2 to interact with cohesin (Figs 2 and 3), we next hypothesized that tumor-derived mutations in *STAG2* might instead lead to generalized abnormalities in the subunit composition of the cohesin complex itself. To test this, we studied previously described isogenic sets of H4 and 42MGBA GBM cells in which the endogenous naturally occurring mutant allele of *STAG2* were corrected by somatic cell gene targeting [5]. Non-detergent nuclear lysates were prepared from these isogenic sets of cells, endogenous cohesin was immunoprecipitated using SMC3 antibodies, and Western blot analysis performed using antibodies specific to the cohesin components SMC1A, SMC3, RAD21, STAG1, STAG2, WAPL, PDS5A, and PDS5B.

**Fig 4:**
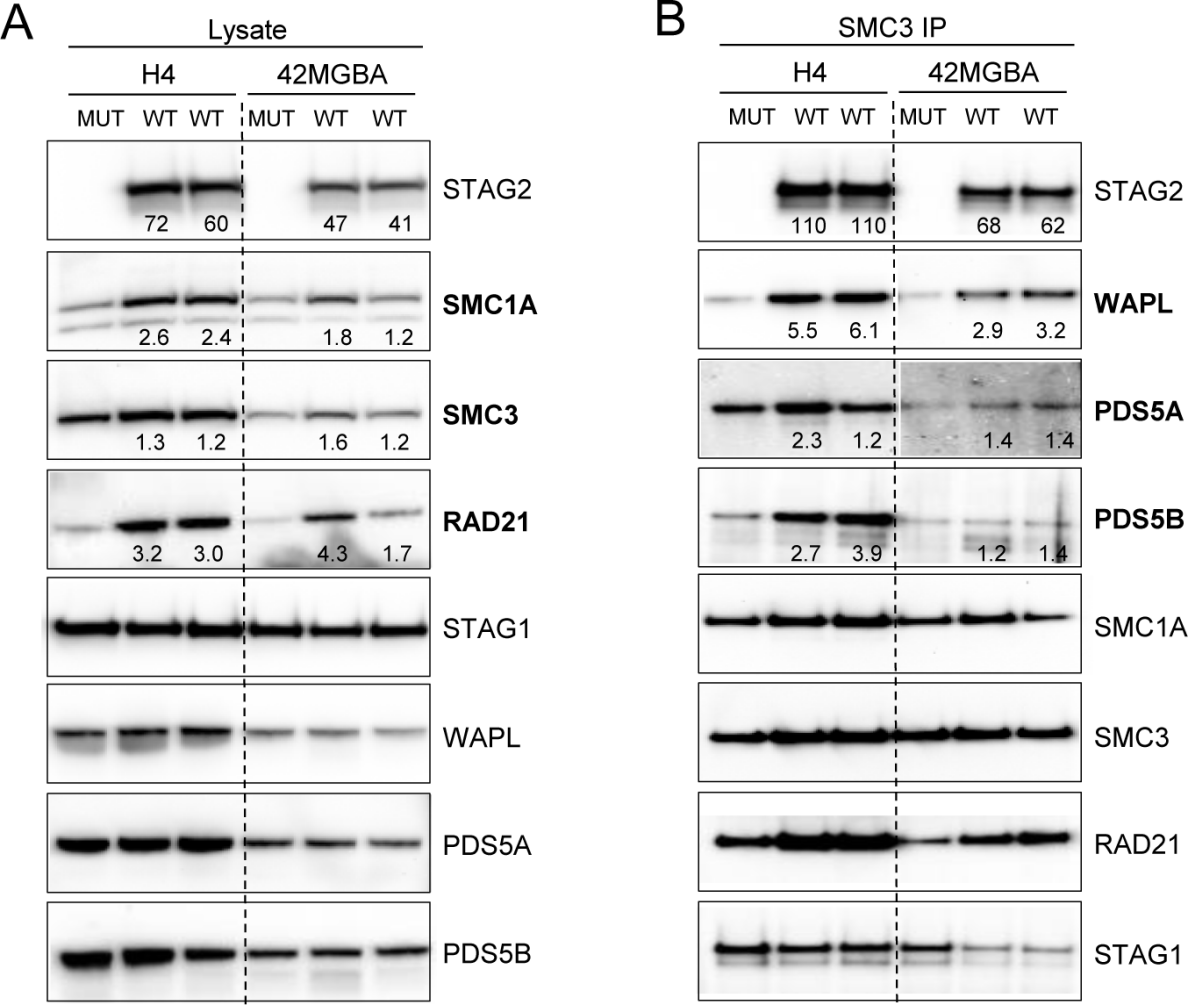
Cohesin subunit composition in *STAG2* mutant human cancer cells. (A) Nuclear lysates derived from isogenic *STAG2* mutant (MUT) and corrected (WT) H4 and 42MGBA GBM cells were studied by Western blot using the antibodies indicated. (B) Nuclear lysates used in (A) were subjected to immunoprecipitation using SMC3 antibodies and Western blots performed with the antibodies indicated. The complete blot with all negative control lanes included is shown in S1 Fig. For both (A) and (B) bands were quantified using ImageJ software, and fold increase compared to isogenic *STAG2* mutant parental cells is indicated below the relevant bands. The results depicted represent a trend that was consistent in four independently-derived clones representing two unrelated sets of isogenic *STAG2* mutant and wild-type cell lines.

Correction of the endogenous mutant allele of *STAG2* led to an increase in total levels of the core cohesin subunits SMC1A, SMC3, and RAD21 in both H4 and 42MGBA cells, but did not affect the total levels of regulatory factors WAPL, PDS5A, or PDS5B (Fig 4A). Targeted correction of mutant *STAG2* did not affect the ability of SMC3 to co-immunoprecipitate SMC1A, SMC3, or RAD21 (Fig 4B; S1 Fig), demonstrating that STAG2 is not required for assembly of the core cohesin ring. However, targeted correction of mutant STAG2 substantially enhanced the ability of WAPL, and to a lesser extent PDS5A and PDS5B, to interact with cohesin (Fig 4B), consistent with recently published structural studies showing that STAG2 functions in part as a structural scaffold for the interaction of WAPL with the core cohesin ring [27]. Taken together, these data indicate that the presence of tumor-derived *STAG2* mutations results in a decrease in levels of cohesin and a reduction in ability of WAPL, PDS5A, and PDS5B to interact with cohesin.

### Modelling of nine tumor-derived *STAG2* mutations in human cells by AAV-mediated human somatic cell gene targeting

We next wanted to determine the functional significance of a representative subset of these mutations in human cells. In an initial attempt to do this, we created several *STAG2* expressing lentiviruses for reconstituting wild-type and mutant *STAG2* expression in cancer cells harboring *STAG2* mutations. However, we found that expression of *STAG2* from these ectopic systems was inconsistent and substantially less than that expressed from the endogenous allele in cells with wild-type *STAG2*.

To circumvent this, AAV-mediated human somatic cell gene targeting was used to “knock-in” (KI) tumor-derived mutations into the endogenous allele of *STAG2* in HCT116 cells, a human cancer cell line with a single, wild-type allele of *STAG2*, intact sister chromatid cohesion, and a near-diploid karyotype. Of note, we have previously used a similar approach to KI a single, non-tumor derived amino terminal nonsense mutation at codon six into *STAG2* in HCT116 cells and demonstrated a substantial reduction in sister chromatid cohesion and alterations in chromosome counts [5].

**Fig 5:**
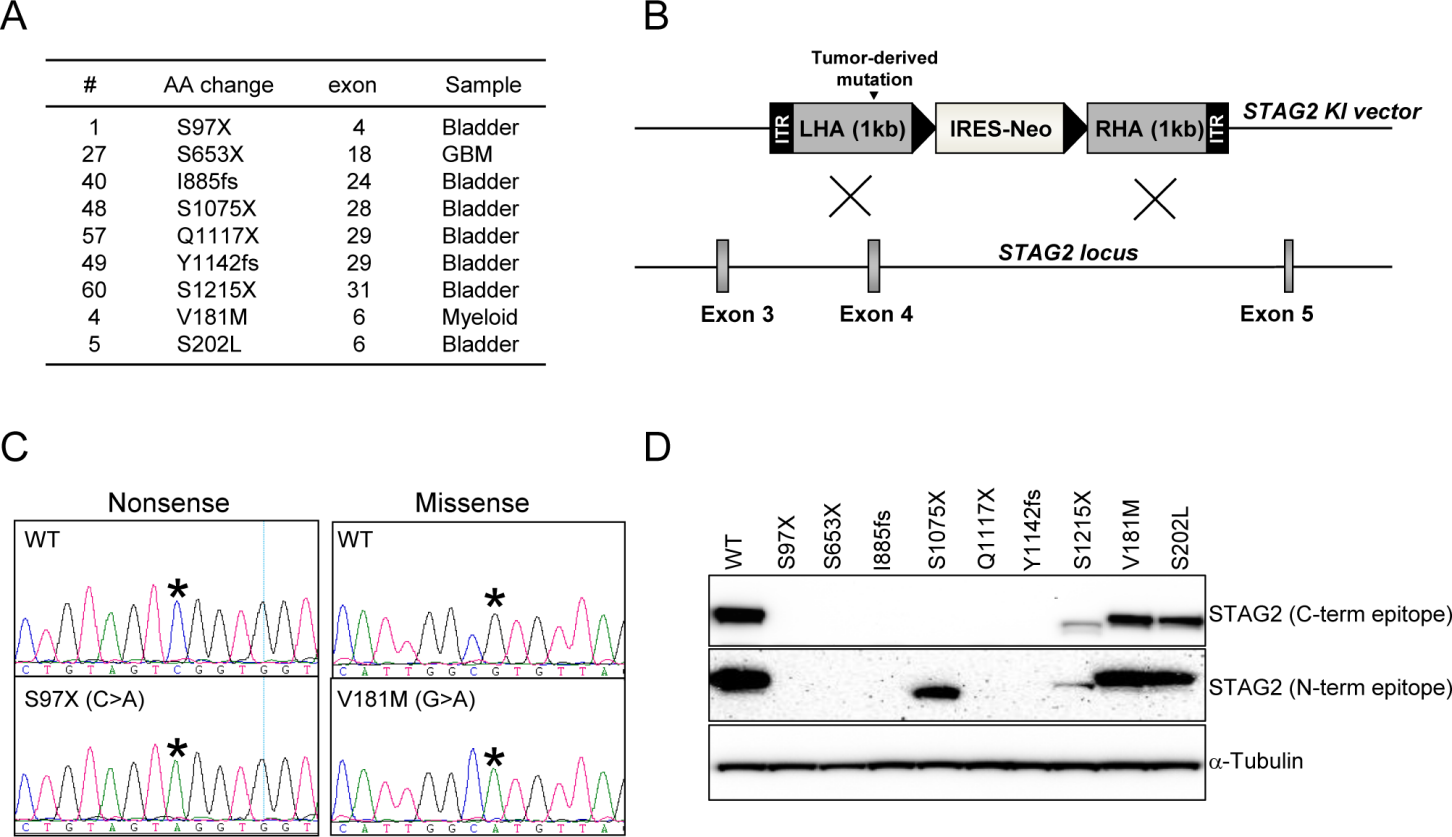
Knockin (KI) of tumor-derived *STAG2* mutations into near-diploid, chromosomally stable cultured human cells. (A) The nine mutations introduced into HCT116 cells. (B) Schematic of AAV-based KI vector for KI of tumor-derived mutations into the endogenous allele of *STAG2*. (C) Representative sequencing traces of parental and KI cells for two of the mutations after targeted integration. (D) STAG2 Western blot for parental and KI cells using antibodies specific to the amino terminus and carboxyl terminus of the STAG2 protein.

To do this, we created nine AAV-based targeting vectors in the pAAV-SEPT vector system and used these vectors to create clonal derivatives of HCT116 cells in which the single endogenous allele of *STAG2* had been mutated (Fig 5A–5C). The details of this approach are described in Materials and Methods. Targeting efficiencies were 14–29% (S2 Table). Western blot analysis with a monoclonal antibody recognizing the carboxyl terminus of STAG2 demonstrated that, as expected, cells harboring the seven nonsense mutations completely lacked expression of the c-terminal epitope (Fig 5D). In contrast, the two missense mutations were expressed at levels equivalent to wild-type *STAG2*. Western blotting with an antibody recognizing the amino terminus of STAG2 further demonstrated that the truncated proteins were expressed poorly, concordant with the ectopic expression data shown in Fig 1 and consistent with the notion that the transcripts may have been degraded via nonsense-mediated decay.

### Effects of endogenous *STAG2* mutations on cellular proliferation

**Fig 6:**
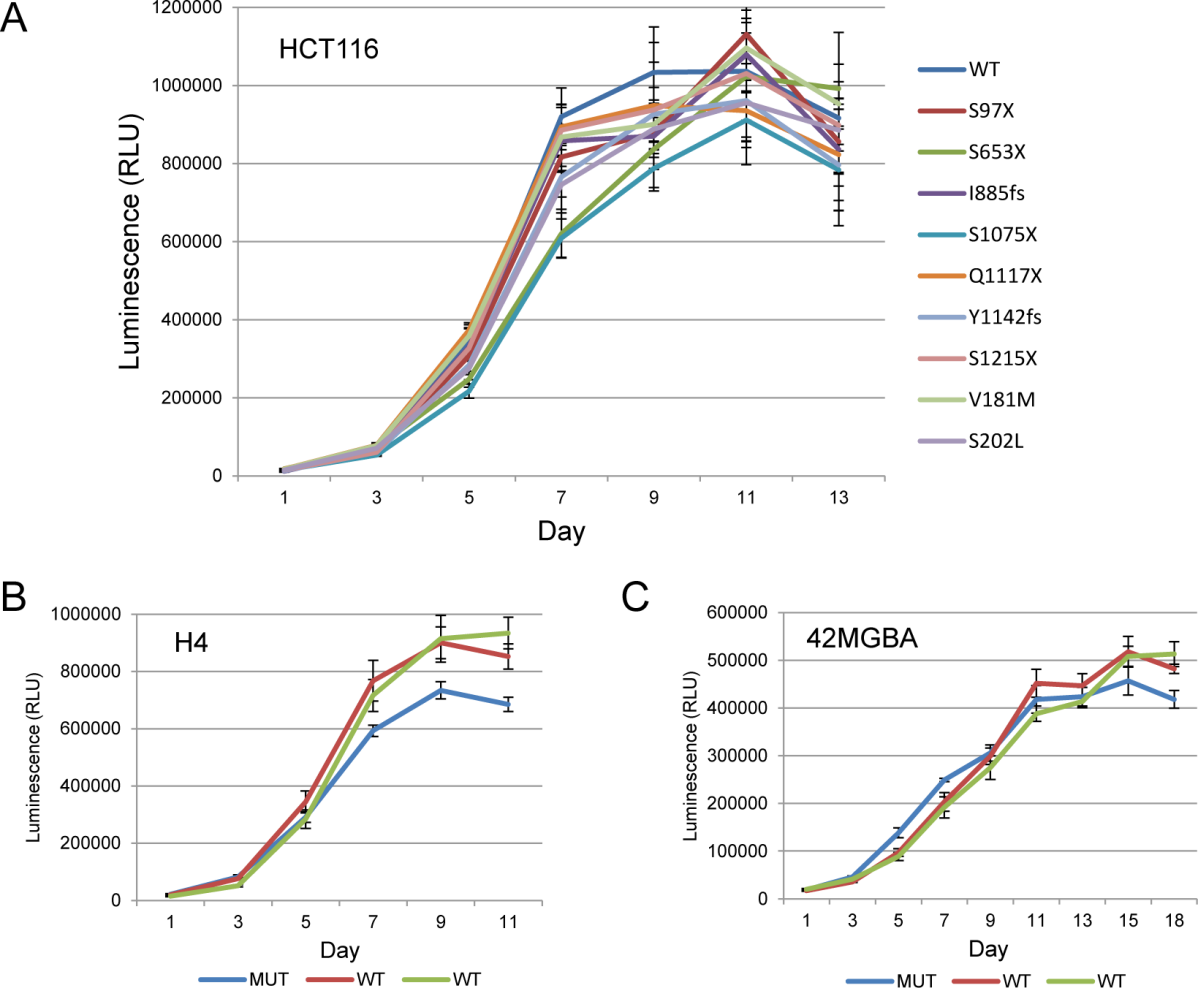
Proliferation assays of isogenic *STAG2* wild-type and mutant cell lines. Cell proliferation was measured using the Cell Titre Glo assay for (A) HCT116 cells and KI derivatives harboring tumor-derived mutations of *STAG2*, (B) H4 cells (MUT) and *STAG2*-corrected derivatives (WT), and (C) 42MGBA cells (MUT) and *STAG2*-corrected derivatives (WT). Though the differences in proliferation between isogenic *STAG2* wild-type and mutant cells were subtle and did not reach p<0.05, the observation that *STAG2* wild-type cells proliferate more quickly than otherwise isogenic *STAG2* mutant cells was a trend that was seen in three different isogenic sets of cell lines (shown in A, B, and C), and in each case seen in multiple independently-derived gene targeted clones.

To determine whether the introduction of tumor-derived mutations had an effect on the proliferation of HCT116 cells, in vitro cellular proliferation assays were performed on the isogenic sets of cells. As shown in Fig 6A, mutation of *STAG2* had a slightly adverse effect on the proliferation of HCT116 cells. To confirm and extend this result, we performed similar assays on two isogenic sets of GBM cells (42MGBA, H4) in which the endogenous mutant allele of *STAG2* had been corrected by gene targeting. As predicted by the result in the HCT116 cell lines, in both H4 cells and 42MGBA cells, correction of *STAG2* led to slightly enhanced proliferation (Fig 6B and 6C). Taken together, these data suggest that unlike in the case of most tumor suppressor genes, mutational inactivation of *STAG2* may have an adverse effect on proliferation.

### Effects of nonsense and missense mutations in *STAG2* on sister chromatid cohesion in human cells

**Fig 7:**
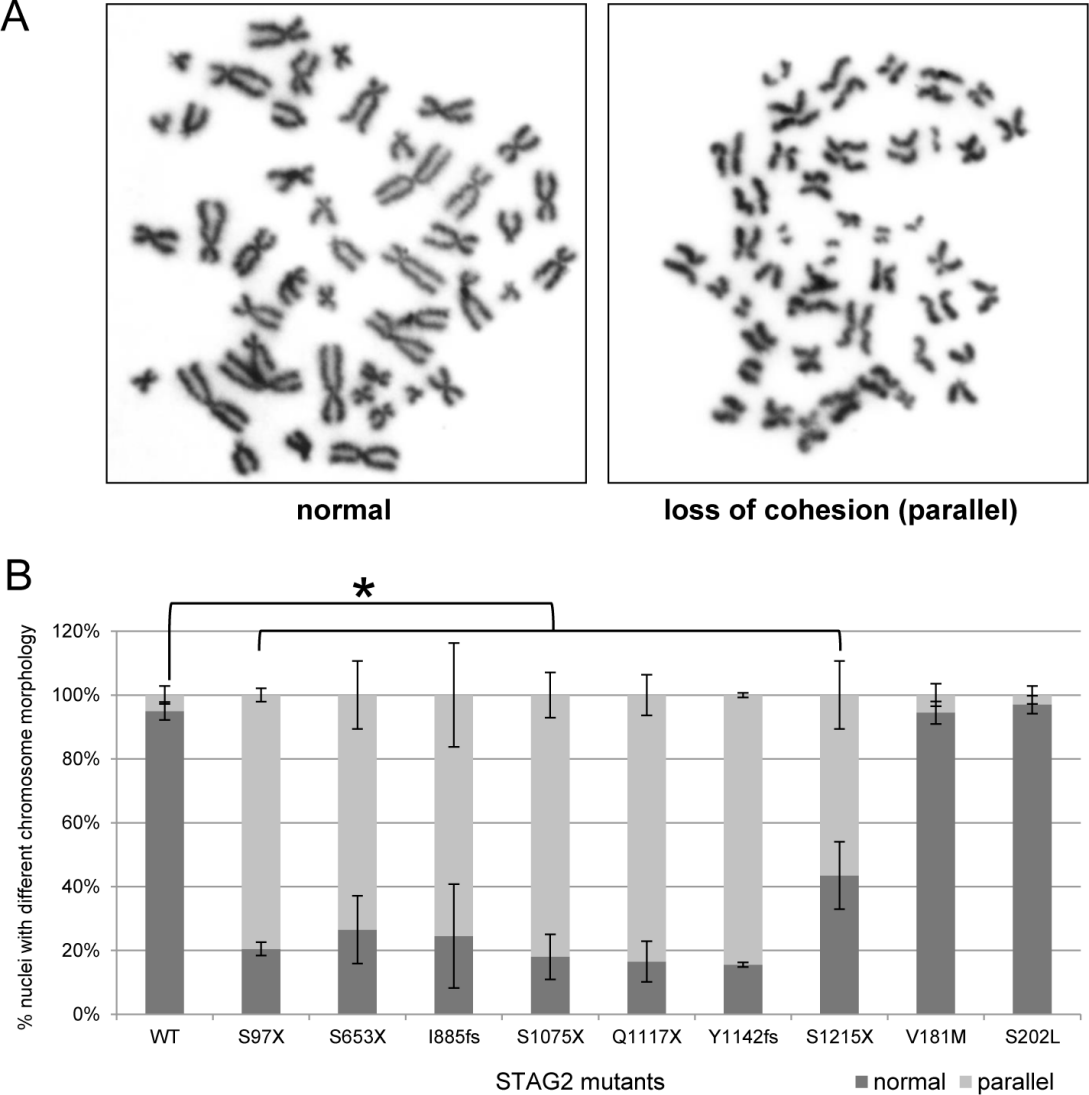
Sister chromatid cohesion assays. HCT116 cells and *STAG2* mutant derivatives were enriched in prometaphase by brief treatment with nocodazole, and chromosome spreads were prepared and analyzed by light microscopy (100X objective) to determine whether mutations led to aberrations in cohesion. Examples are shown in (A), and quantification is shown in (B). A cell was determined to have loss of cohesion if three or more sister chromatids were parallel. 100 prometaphases were counted per cell line. Counting was performed in a blinded fashion. The differences in numbers of parallel sister chromatids between parental *STAG2* wild-type cells and derivatives with truncating mutations of *STAG2* were significant (*, p<0.05).

To measure the effects of tumor-derived nonsense and missense mutations on sister chromatid cohesion, we enriched the HCT116 cells and *STAG2* KI derivatives in mitosis by short treatment with nocodazole, then examined prometaphase chromosome spreads to analyze sister chromatid cohesion. For details, see Materials and Methods. As shown in Fig 7, all tumor-derived nonsense mutations in *STAG2* led to a reduction in the integrity of sister chromatid cohesion. In contrast, tumor-derived missense mutations displayed wild-type sister chromatid cohesion. These data suggest either that *STAG2* nonsense mutations have a different pathogenic mechanism than *STAG2* missense mutations, or that abrogation of sister chromatid cohesion is not the key property targeted by tumor-derived mutations in *STAG2*.

### Effects of nonsense and missense mutations in *STAG2* on anaphase integrity in human cells

**Fig 8:**
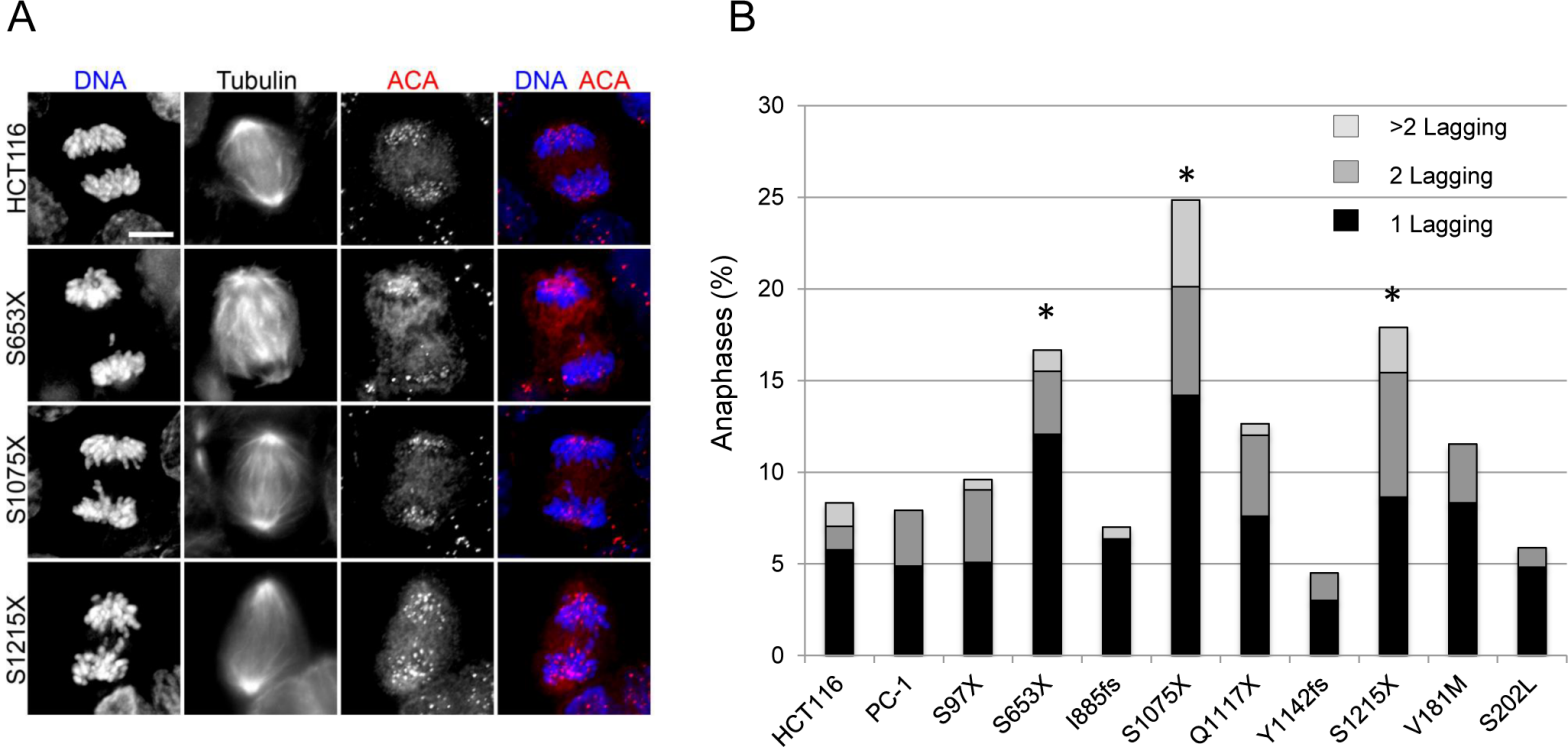
Chromosome segregation during anaphase in *STAG2* mutant cells. Proliferating HCT116 cells and *STAG2* mutant derivatives were triple stained using DAPI and using antibodies to the centromere (ACA) and microtubules (α-tubulin). PC-1 cells are an HCT116 clone with random integration of an AAV targeting vector, and serve as an additional negative control. Anaphase cells were identified and lagging chromosomes and multipolar mitoses were counted. Examples of lagging chromosomes are shown in (A), and quantification is shown in (B). Cells harboring the S653X, S1075X, and S1215X mutations had a statistically significant increase (*, p<0.05) in lagging chromosomes. For raw data, see S3 Table.

Next, we tested whether cells harboring tumor-derived mutations in *STAG2* showed a reduction in the integrity of sister chromatid segregation during anaphase. To do this, proliferating cells were fixed, triple stained with DAPI and antibodies to α-tubulin and the centromere (ACA), and the fractions of anaphase cells with chromosome missegregation errors was determined (for details, see Materials and Methods). Three of seven cell lines harboring *STAG2* truncating mutations (S653X, S1075X, and S1215X) had a statistically significant increase in lagging chromosomes compared to isogenic cells with wild-type *STAG2* (Fig 8; S3 Table). There was no statistically significant increase in the fractions of cells with DNA bridges, acentric fragments, or multipolar anaphases in any of the *STAG2* mutant cells (S3 Table).

### Effects of nonsense and missense mutations in *STAG2* on chromosome counts in human cells

In two previous studies, we found that introduction of a non-tumor derived mutation or stable lentiviral depletion of wild-type *STAG2* led to alterations in chromosome counts in human cells–in both cases increasing the modal chromosome number by one [5,6]. To determine if similar results would be obtained after introduction of tumor-derived mutations into *STAG2*, chromosomes were counted in parental HCT116 cells and derivatives with each of the nine tumor-derived mutations. As described in Materials and Methods, prometaphase chromosome spreads were prepared, and the number of chromosomes in 100 cells was determined for each cell line. Surprisingly, there was no change in modal chromosome number after introduction of eight of the tumor-derived mutations, with the exception of the introduction of S653X, which led to an increase in the modal chromosome number by one (Table 1). This experiment indicates that many tumor-derived mutations in *STAG2* do not have an overt effect on chromosome count when introduced into HCT116 cells.

**Table 1:** Chromosome counts in parental and STAG2 mutant HCT116 cells.

	HCT116	PC-1	PC-2	PC-3	S97X	S653X	I885fs	S1075X	Q1117X	Y1142fs	S1215X	V181M	S202L
**39**			2										
**40**			1	1					1				
**41**								1	2				
**42**			1	1					3				
**43**	1		4				4	5					
**44**	4	4	12	12	6	1	6	28	12	11	2	2	2
**45**	70*	78*	63*	73*	56*	7	74*	53*	60*	63*	81*	73*	78*
**46**	22	18	16	11	38	92*	16	11	21	26	17	25	19
**47**	3		1	2				2	1				1

Chromosomes were counted in 100 prometaphase cells for each cell line. PC-1, PC-2, and PC-3 are three independently derived control clones with random integration of the KI vector. Modal chromosome numbers are indicated with asterisk.

## Discussion

Somatic mutations of genes encoding components of the cohesin complex are common in a variety of adult and pediatric human cancers. The most commonly mutated cohesin gene is *STAG2*, with other cohesin genes such as *RAD21*, *SMC1A* and *SMC3* also mutated in a subset of tumors. Now that the frequencies and tumor-type specificities of these mutations have been established, it has become increasingly important to determine the functional role played by these mutations in tumorigenesis. In the studies presented here we have focused our attention on the role of tumor-derived mutations in *STAG2* on the protein composition of cohesin, cellular proliferation, mitosis, and aneuploidy.

Arguably the most straightforward hypotheses regarding the loss of function caused by tumor-derived *STAG2* mutations is that the mutations either abolish the ability of STAG2 to interact with the rest of the cohesin complex, or that they lead to more generalized abnormalities in the subunit composition of cohesin. To test these hypotheses, we expressed 59 tumor-derived mutations in human cells in an epitope tagged *STAG2* cDNA. Many of the mutations led to virtually complete loss of expression of the encoded protein, consistent with the expectation that *STAG2* genes harboring tumor-derived nonsense mutations produce transcripts that are degraded by nonsense mediated decay. We then performed IP-Western blot analyses on the mutant STAG2 proteins that were expressed, and showed that many tumor-derived mutations do not abolish the ability of the encoded protein to interact with cohesin. These data indicate that the ability to interact with the rest of the cohesin complex is not—or at least not the only—key property of STAG2 abrogated by tumor-derived mutations in the gene.

We then tested an alternate hypothesis—that *STAG2* mutations cause alterations in the amount and/or subunit composition of cohesin in human cancer cells. We found that cells harboring *STAG2* mutations have roughly half as much SMC1A, SMC3, and RAD21 as otherwise isogenic cells with wild-type *STAG2*. This result is consistent with previous studies showing that myeloid leukemias with *STAG2* mutations have less chromatin-bound cohesin than leukemias with wild-type *STAG2* [10]. Our result suggests that this may have been due to a reduction in cohesin itself rather than a reduction in the efficiency by which cohesin binds chromatin.

Next, we tested the hypothesis that mutations in *STAG2* result in alterations in the subunit composition of cohesin. We found that *STAG2* mutations did not affect the composition of the core cohesin ring (composed of SMC1A, SMC3, RAD21, and STAG1 or STAG2). However, the presence of a *STAG2* mutation did adversely affect the ability of cohesin regulatory factor WAPL (and to a lesser extent PDS5A and PDS5B) to interact with the core cohesin ring. These results are consistent with current models of cohesin structure, which suggest that STAG2 is not itself a key structural scaffold for assembly of the core cohesin ring, but instead is tethered to cohesin via its interaction with RAD21. Furthermore, our finding that interaction of WAPL with cohesin depends in part on STAG2 is in agreement with recently published structural studies showing that STAG2 functions as a structural scaffold for the interaction of WAPL with the core cohesin ring [27]. Of note, WAPL is required for removal of cohesin from chromosome arms during prophase, and reductions in cohesin-bound WAPL have previously been shown to lead chromosomal non-disjunction and aneuploidy [28].

Next, we used human somatic cell gene targeting to introduce a subset of these tumor-derived mutations into the endogenous allele of *STAG2* in a chromosomally stable, near diploid human cell line. Despite the fact that only 14% of tumor-derived mutations in *STAG2* are missense (the remainder are truncating), we intentionally introduced several missense mutations since we believe their effects are likely to be more subtle and therefore could shed important light on the specific functional deficiencies of STAG2 in cancer. Interestingly, genetically modified cells harboring *STAG2* mutations proliferated slightly more slowly than isogenic parental cells with wild-type *STAG2* –a result which was then confirmed by measuring the proliferation of several additional isogenic sets of GBM cells in which the endogenous mutant allele of *STAG2* had been corrected by human somatic cell gene targeting.

This observation that endogenous *STAG2* mutations slow cellular proliferation is surprising, since mutations in tumor suppressor genes are generally thought to enhance cellular proliferation. Of note, our proliferation data are in disagreement with recent results from Balbas-Martinez et al. and Kon et al., who showed that ectopic re-expression of wild-type *STAG2* and RAD21 in cancer cells harboring endogenous mutations of those genes leads to suppression of proliferation [8,10]. It is possible that the discrepancies between our data and those of Balbas-Martinez and Kon et al. are due to differences between ectopic and endogenous expression of wild-type *STAG2* and/or cell type specific differences.

Using these isogenic sets of cells, we next tested the effect of tumor-derived nonsense and missense mutations in *STAG2* on sister chromatid cohesion, mitotic fidelity, and chromosome counts. As expected based on our previous studies, all seven nonsense mutations in *STAG2* led to substantial reductions in the integrity of sister chromatid cohesion during metaphase. However, to our surprise, tumor-derived missense mutations retained wild-type levels of sister chromatid cohesion. Furthermore, only a subset of the nonsense mutations (and none of the missense mutations) caused an increase in lagging chromosomes during anaphase, generally considered a marker of chromosomal instability. Furthermore, despite the substantial defects in cohesion in cells with *STAG2* nonsense mutations (and the increase in lagging chromosomes in a subset of the cell lines), only one of the mutant *STAG2* KI cell lines demonstrated alterations in chromosome counts when compared to isogenic cells with wild-type *STAG2* genes.

These results raise a number of interesting issues and questions regarding the role of *STAG2* gene mutations in cancer pathogenesis. First, we believe that the study of tumor-derived missense mutations provides a particularly useful window into the specific functions of STAG2 that are relevant to cancer pathogenesis, since the adverse effects of missense mutations are likely to more specific to cancer pathogenesis than the more pleiotropic effects of early truncating mutations (with the caveat that it is a formal possibility that a subset of missense mutations are passenger mutations). The fact that the missense mutations tested retained wild-type cohesion, mitotic integrity, and ploidy suggests that perhaps these phenotypes are not central to the cancer-causing effects of *STAG2* mutations. Such an interpretation could help resolve a fundamental current discrepancy in the field–that all tumor-derived mutations tested so far (all of which have been truncating mutations) result in dramatic reductions in cohesion, and yet many naturally occurring tumors with *STAG2* mutations appear to be euploid. Alternatively, it is a formal possibility that nonsense and missense mutations in *STAG2* lead to deficiencies in two different functions of STAG2 and therefore lead to cellular transformation through two different pathways. However, we consider this possibility to be unlikely.

The chromosome counting data presented here are especially surprising in light of our previously published data showing that the introduction of an amino terminal, non-tumor derived truncating mutation into *STAG2* leads to an increase in modal chromosome number [5]. Similar results were also obtained in a bladder cancer cell line with shRNA-mediated depletion of *STAG2* [6]. It is particularly surprising that only one of the seven nonsense mutations tested resulted in an alteration in chromosome count, despite the fact that all nonsense mutations led to substantial reductions in sister chromatid cohesion and a subset led to an increase in lagging chromosomes during anaphase. Though surprising in light of the preponderance of published data on the relationship of cohesion to aneuploidy, these data are consistent with published work in *Saccharomyces cerevisiae* suggesting that low levels of cohesin can be sufficient to maintain euploidy [29,30]. When taken together with the results presented for the missense mutations tested, these results further call into question whether the cancer relevant phenotypes of *STAG2* mutations are directly related to cohesion and aneuploidy.

In summary, here we analyze the properties of a substantial number of tumor-derived mutations in *STAG2*. In addition to evaluating the effect of the mutations on the composition of cohesin, we demonstrate that the mutations do not uniformly abrogate sister chromatid cohesion, anaphase integrity, or the ability of the cells to control their ploidy. These data suggest either that different mutations of *STAG2* have different mechanisms through which they cause cancer, or that the specific mechanism(s) through which *STAG2* and other cohesin gene mutations contribute to cancer pathogenesis remain, at present, unknown.

## Materials and Methods

### Human *STAG2* expression vector

A human *STAG2* cDNA (CCDS43990) with an amino terminus 1X FLAG/SBP dual epitope tag was synthesized de novo by Genscript and cloned into pUC57. Tumor-derived mutations were created by site-directed mutagenesis using the QuikChange II XL kit (Stratagene) as directed by the manufacturer. Wild-type and mutant cDNAs were then subcloned into pcDNA3.1.

### Cell culture

HCT116 cells were obtained from ATCC. H4 and 42MGBA parental cells and gene targeted derivatives in which the endogenous mutant allele of *STAG2* was corrected by AAV-mediated human somatic cell gene targeting were described previously [5].

### Antibodies

Primary antibodies for immunoblotting were FLAG (M2) from Sigma Aldrich; STAG2 (J-12; carboxyl-terminus epitope), STAG1 (A-9), RAD21 (B-2), SMC3 (E-3), SMC1A (M-16), WAPL (A-7), and PDS5A (S-20) from Santa Cruz Biotechnology; and PDS5B (A300-537A) and STAG2 (A302-580A; amino terminus epitope) from Bethyl Laboratories. Antibodies and conjugated beads for immunoprecipitation were FLAG-M2 beads from Sigma Aldrich, SMC3 (A300-060A) from Bethyl Laboratories, and Streptavidin Plus UltraLink Resin from Pierce. Antibodies for immunofluorescence were anti-centromere antibody (ACA) from Geisel School of Medicine, α-tubulin (DM1α) from Sigma Aldrich, and Alexa Fluor 488 and 568 from Molecular Probes.

### Immunoprecipitation and western blot

Protein lysates for direct Western blotting were prepared in NP40 lysis buffer (50 mM Tris-HCl pH 7.5, 150 mM NaCl, 1% NP40). Nuclear lysates used for affinity purification were prepared using a modification of Dignam's nondetergent lysis method [31]. Protein concentrations were determined using the bicinchoninic assay (Pierce). For affinity purification of proteins tagged with Streptavidin Binding Peptide (SBP), streptavidin beads (Pierce) were washed once with Tris-buffered saline (TBS; 150 mM NaCl) and then incubated with protein lysates derived from cells transfected with constructs expressing epitope-tagged proteins. Samples were incubated with rotation at 4°C for 1 h. Beads were then washed three times with TBS, and bound proteins eluted by boiling in sample buffer, separated by SDS-PAGE, and Western blot analysis performed.

### AAV-mediated human somatic cell gene targeting

Homology arms (~1 kb each) for creation of *STAG2* KI vectors were synthesized by Genscript and cloned into the pAAV-SEPT-Acceptor vector [32]. Next, transient stocks of AAV-2 virions were created by co-transfection of 293T cells with *STAG2* KI vectors together with pAAV-RC (Stratagene) and pHELPER (Stratagene) using X-tremeGENE 9 (Roche). Two days after transfection, media was aspirated and cell monolayers were scraped into 1 mL PBS and subjected to four cycles of freeze/thaw. The lysate was then clarified by centrifugation at 12,000 rpm for 10 min in a benchtop microfuge to remove cell debris, and the virus-containing supernatant was aliquoted and stored at −80°C. 200 μL of virus was then used to infect HCT116 cells in T25 tissue culture flask, and cells were passaged at limiting dilution into 96-well plates in the presence of 1.0 mg/mL G418. Individual G418-resistant clones were expanded and used for the preparation of genomic DNA. Clones were tested for homologous integration of the targeting vector using a primer pair specific for the targeted allele, and integration of the targeted mutation was confirmed by DNA sequencing. For missense mutations, KI cells were then infected with a Cre-expressing adenovirus to remove the FLOXed splice acceptor-IRES-neo^R^ gene.

### Proliferation assays

Cells were seeded at either 500 or 1000 cells/well in 96 well plates and cell number measured every 48 hours using the Cell Titre Glo Assay (Promega).

### Sister chromatid cohesion assay

Cells were grown to approximately 80% confluence, then treated with 330 μM nocodazole for one hour. Cells were then harvested by trypsinization and hypotonically swollen in 40% medium/60% Vienna tap water for 5 min at room temperature. Cells were fixed with freshly made Carnoy’s solution (75% methanol, 25% acetic acid), and the fixative was changed several times. For spreading, cells in Carnoy’s solution were dropped onto glass slides and dried at 37C. Slides were stained with 5% Giemsa at pH 6.8 for 7 min, washed briefly in tap water, air dried, and mounted with Entellan mounting medium. Each experiment was performed independently twice.

### Immunofluorescence and image processing

Cells were fixed with 3.5% paraformaldehyde (Alfa Aesar) for 10 min, washed with Tris-buffered saline with 5% bovine serum albumin (TBS-BSA) and 0.5% Triton X-100 for 2 x 5 min with vigorous shaking and then rinsed in TBS-BSA. Primary antibodies were diluted in TBS-BSA containing 0.1% Triton X-100 and incubated for 1–3 h at room temperature. Cells were then washed with TBS-BSA/0.1% Triton X-100 for 3 x 10 min with vigorous shaking. Secondary antibodies were diluted in TBS-BSA plus 0.1% Triton X-100 and coverslips were incubated for 1–2 h at room temperature. Cells were then washed with TBS-BSA containing 0.1% Triton X-100 with DAPI for 3 x 10 min with vigorous shaking and then mounted using ProLong Gold antifade reagent (Molecular Probes). Images were acquired with a cooled charge-coupled device camera (Andor Technology, Belfast, UK) mounted on a Nikon Eclipse Ti microscope (Nikon, Melville, NY) with a 60X, 1.4 numerical aperture objective. Image series in the *z*-axis were obtained using 0.2-μm optical sections. Image deconvolution and contrast enhancement was performed using AutoQuant X3 (Media Cybernetics), Elements software (Nikon), Image J and Adobe Photoshop software. Final images represent selected overlaid planes.

For quantifications of anaphase segregation defects, chromatids were counted as lagging if they contained centromere staining (using ACA) or acentric if they did not contain centromere staining, in the spindle midzone separated from centromeres/kinetochores at the poles. DNA bridges were counted when a piece of stretched DNA (visualized by DAPI staining) spanned the area between the two newly formed daughter nuclei in anaphase and no centromere staining was evident. Tripolar anaphases were counted when three clear chromosome populations (with centromere staining) were evident in anaphase. For quantification of anaphase chromosome missegregation rates, no fewer than 150 anaphases per cell line were quantified. Statistical analysis for anaphase chromosome missegregation rates was performed using Fisher’s exact two-tailed test.

### Chromosome counting

Cultured cells were treated with 0.02 μg/ml colcemid for 45 minutes at 37°C. The cells were then trypsinized, centrifuged at 200 x g, and the cell pellet resuspended in warmed hypotonic solution and incubated at 37°C for 11 minutes. The swollen cells were then centrifuged and the pellet resuspended in 8 mL of Carnoy’s fixative (3:1 methanol:glacial acetic acid). After incubation in fixative at room temperature for 22 minutes, the cell suspension was centrifuged and washed twice in Carnoy’s fixative. After the last centrifugation, the cells were resuspended in 1 to 3 mL freshly prepared fixative to produce an opalescent cell suspension. Drops of the final cell suspension were placed on clean slides and air-dried. Slides were stained with a 1:3 mixture of Wright’s stain and 60 mM phosphate buffer for 4–10 minutes, washed with tap water for 5 seconds, and then air-dried. Chromosomes were counted in 100 prometaphases per cell line.

## Supporting Information

S1 Fig Cohesin subunit composition in *STAG2* mutant human cancer cells. The blot shown is identical to that shown in Fig 4 except with all negative control IgG IP lanes shown
(TIFF)

S1 Table *STAG2* mutations analyzed in this study. (TIFF)

S2 Table Targeting efficiencies for the human somatic cell targeting vectors used in this study. (TIFF)

S3 Table Effects of *STAG2* mutations on anaphase integrity. (TIFF)
