# Supplementary figures and images for: Intact Cohesion, Anaphase, and Chromosome Segregation in Human Cells Harboring Tumor-Derived Mutations in STAG2
Source: PLoS Genet. 2016 Feb 12;12(2):e1005865. doi: 10.1371/journal.pgen.1005865 (PMC4752446; doi:10.1371/journal.pgen.1005865)

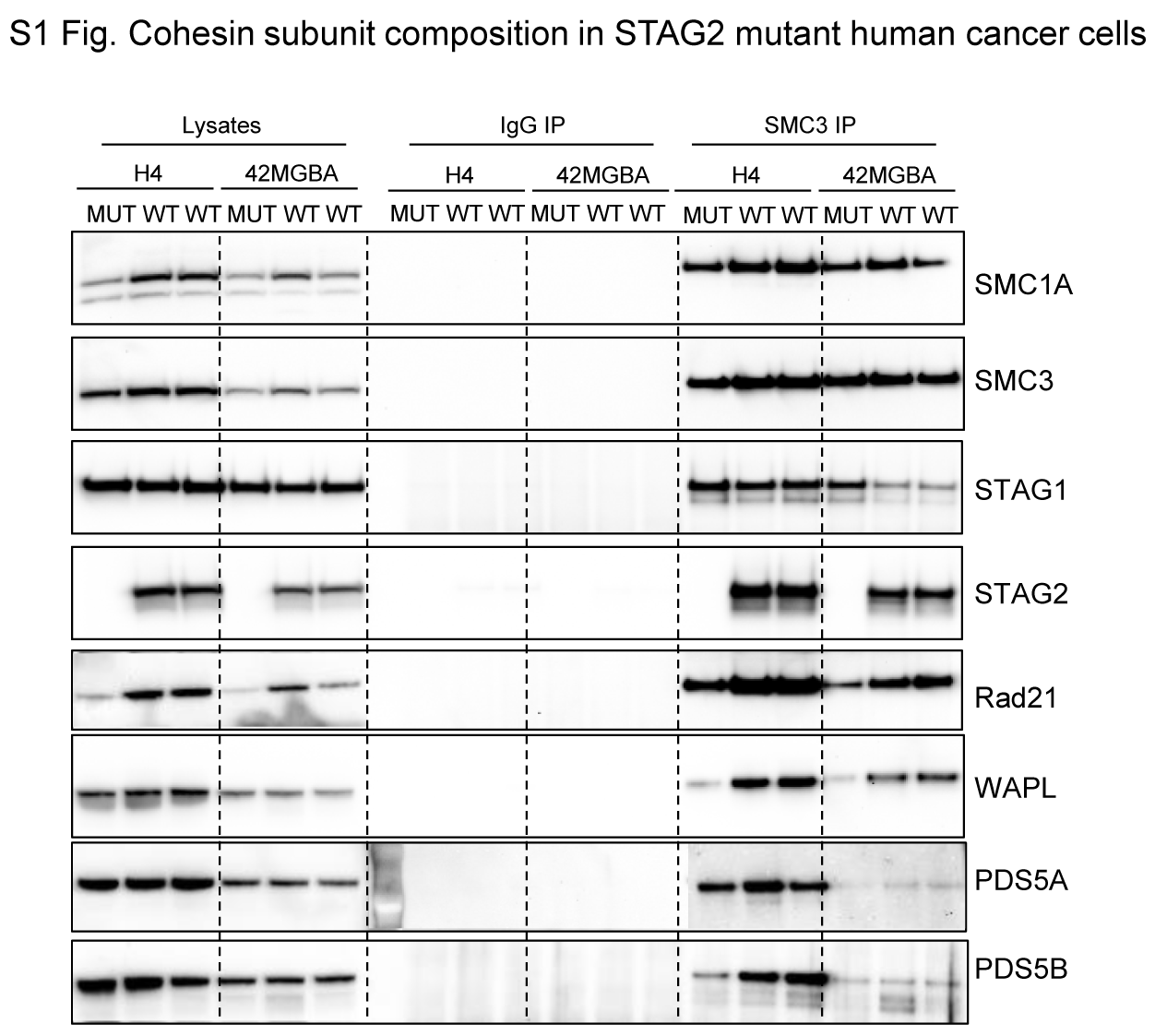

Supplement: S1 Fig — The blot shown is identical to that shown in Fig 4 except with all negative control IgG IP lanes shown (TIFF) [file pgen.1005865.s001.tiff]

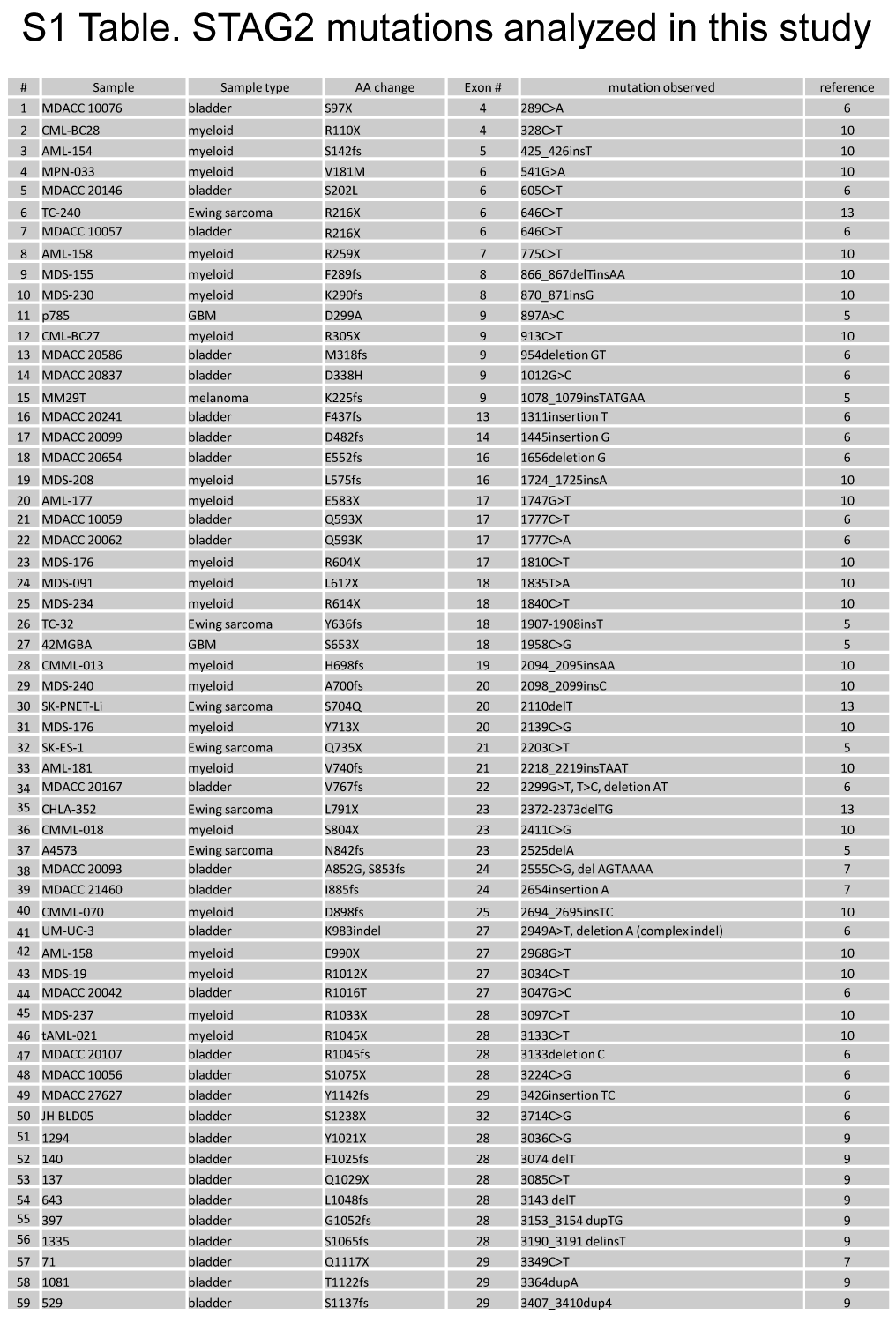

Supplement: S1 Table — (TIFF) [file pgen.1005865.s002.tiff]

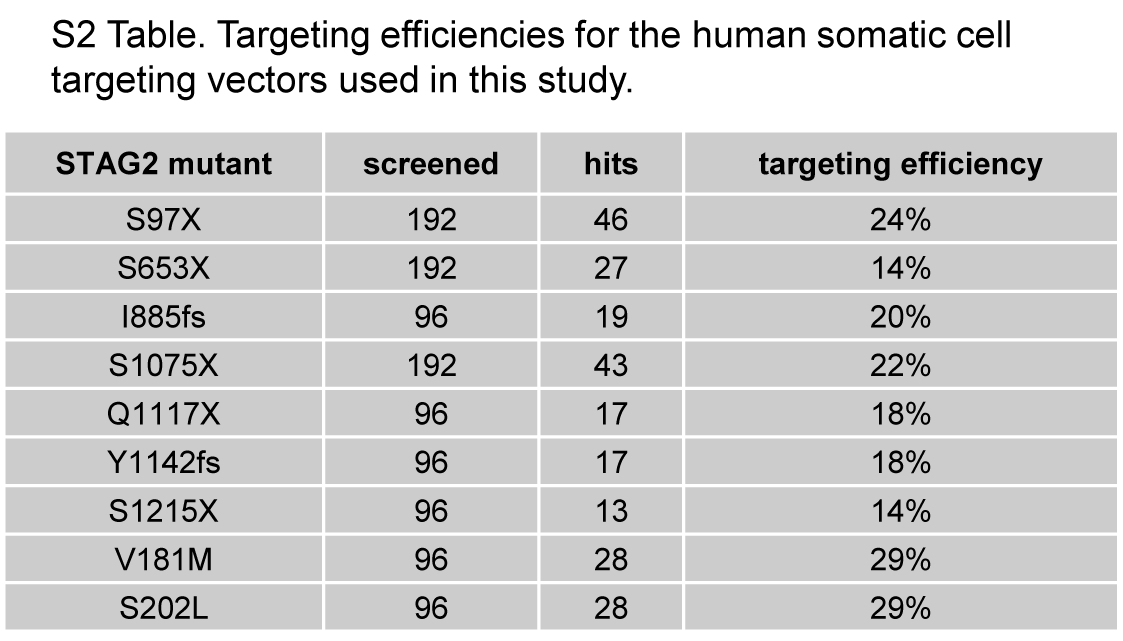

Supplement: S2 Table — (TIFF) [file pgen.1005865.s003.tiff]

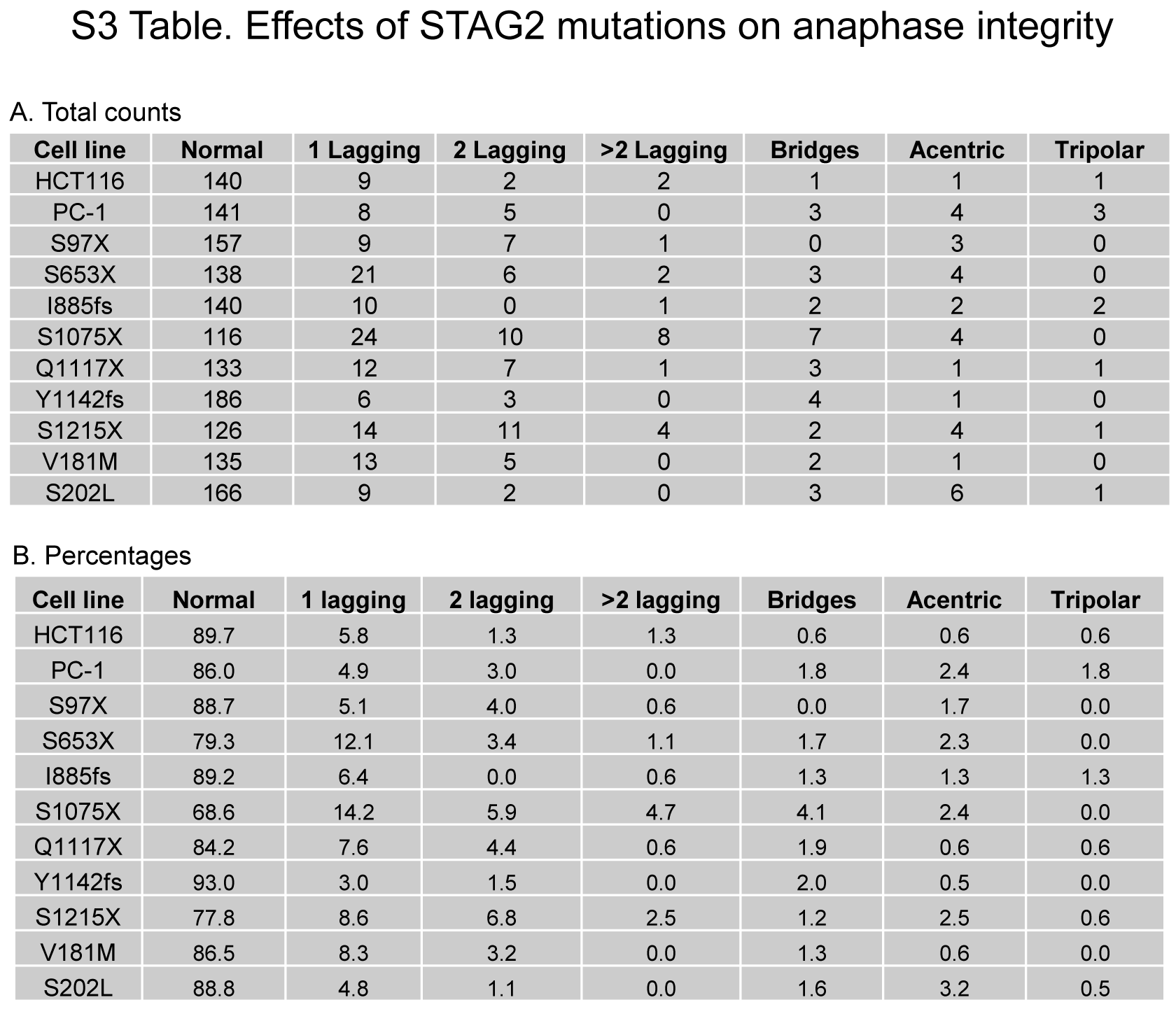

Supplement: S3 Table — (TIFF) [file pgen.1005865.s004.tiff]
